# Coordinated Hsp110 and Hsp104 Activities Power Protein Disaggregation in Saccharomyces cerevisiae

**DOI:** 10.1128/MCB.00027-17

**Published:** 2017-05-16

**Authors:** Jayasankar Mohanakrishnan Kaimal, Ganapathi Kandasamy, Fabian Gasser, Claes Andréasson

**Affiliations:** Department of Molecular Biosciences, The Wenner-Gren Institute, Stockholm University, Stockholm, Sweden

**Keywords:** Hsp110, Sse1, Sse2, Hsp104, Ssa1, protein disaggregation, chaperone, protein misfolding, heat shock, protein folding, protein quality control, stress proteins

## Abstract

Protein aggregation is intimately associated with cellular stress and is accelerated during aging, disease, and cellular dysfunction. Yeast cells rely on the ATP-consuming chaperone Hsp104 to disaggregate proteins together with Hsp70. Hsp110s are ancient and abundant chaperones that form complexes with Hsp70. Here we provide *in vivo* data showing that the Saccharomyces cerevisiae Hsp110s Sse1 and Sse2 are essential for Hsp104-dependent protein disaggregation. Following heat shock, complexes of Hsp110 and Hsp70 are recruited to protein aggregates and function together with Hsp104 in the disaggregation process. In the absence of Hsp110, targeting of Hsp70 and Hsp104 to the aggregates is impaired, and the residual Hsp104 that still reaches the aggregates fails to disaggregate. Thus, coordinated activities of both Hsp104 and Hsp110 are required to reactivate aggregated proteins. These findings have important implications for the understanding of how eukaryotic cells manage misfolded and amyloid proteins.

## INTRODUCTION

Protein aggregates are hallmarks of stressed cells and accumulate under conditions of disease and aging (1, 2). During acute folding stress, misfolded proteins are routed from the cytoplasm to insoluble protein aggregates. The aggregation process is spontaneous but is also guided by specialized aggregation factors that interact with the misfolded proteins (3–5). Such orchestrated aggregation is believed to protect cells from the acute proteotoxicity of misfolded species and to provide a buffer for the downstream systems that manage misfolded proteins.

An intricate network of ATP-consuming chaperones with disaggregation and refolding activities disentangles and reactivates aggregated proteins (6). Most organisms, including bacteria, plants, and fungi, employ a bichaperone system comprised of interacting ATP-dependent chaperones of the Hsp70 and Hsp100 classes to perform protein disaggregation (6, 7). Accordingly, in Saccharomyces cerevisiae, the Hsp70 cochaperone Hsp40 associates with the surface of the aggregates and recruits Hsp70. Hsp70 locks onto exposed hydrophobic amino acid stretches of the aggregated proteins by undergoing conformational changes controlled by ATP hydrolysis. Next, ADP-bound Hsp70 recruits the ring-shaped hexameric disaggregase Hsp104 that translocates the protein substrate through its central pore channel. The force-generating ATPase activity of Hsp104 that drives translocation is stimulated by interactions with Hsp70. Once translocated through Hsp104, the disentangled protein folds into its native conformation.

Metazoan cells do not possess Hsp104-encoding genes and instead rely on Hsp70s and their relatives Hsp110s for protein disaggregation. Together, Hsp40s, Hsp70s, and Hsp110s form dynamic complexes that disentangle and reactivate aggregated proteins *in vitro* (8–12). In this process, the substrate and Hsp40 synergistically stimulate Hsp70 ATPase activity, which results in the trapping of the substrate by Hsp70-ADP. Hsp110 binds to Hsp70-ADP and thereby accelerates nucleotide exchange and triggers the release of Hsp70-associated substrates (13–16). Studies have arrived at different conclusions as to whether Hsp110 depends on its ATPase activity to accelerate protein disaggregation *in vitro* (8, 10, 11). *In vivo* evidence from genetic experiments with heat-shocked Caenorhabditis elegans supports the notion that Hsp110 plays an important role in metazoan protein disaggregation (10). Intriguingly, the overexpression of Hsp110 has been shown to ameliorate neurodegeneration associated with cytosolic misfolding and aggregation in mice that express mutant Cu/Zn superoxide dismutase 1 (17). However, the design of those experiments with ongoing translation raises the question of whether Hsp110 genuinely acts on bona fide aggregated proteins or exerts its effects by participating in the Hsp70-dependent folding of newly translated proteins (18). Thus, Hsp110-dependent disaggregation awaits unequivocal *in vivo* demonstration.

In yeast, the reactivation of aggregated proteins is strictly dependent on Hsp104, and the involvement of the yeast Hsp110s Sse1 and Sse2 is unclear. Reducing Hsp110 expression by genetically removing either *SSE1* or *SSE2* does not impair Hsp104-dependent reactivation of heat-aggregated firefly luciferase (FFL) (19). However, upon the complete genetic removal of the essential Hsp110 (*sse1*Δ *sse2*Δ), which was possible only with strong overexpression of the Hsp70 nucleotide exchange factor Fes1, the refolding of thermally denatured proteins was delayed (15). *In vitro*, reconstituted Hsp104-dependent disaggregation has been demonstrated to function in a minimal purified system consisting of the yeast Hsp70 Ssa1 and yeast Hsp40s (20). The addition of Sse1 to such reactions accelerated the Hsp104-dependent reactivation of chemically aggregated firefly luciferase (11). A similar acceleration of Hsp104-dependent disaggregation was observed when employing firefly luciferase aggregated together with the small heat shock protein Hsp26 during thermal denaturation (10). Thus, while data from *in vitro* experiments suggest that Hsp110 has the potential to accelerate Hsp104-dependent disaggregation, convincing *in vivo* evidence for such a role is lacking.

Here we present *in vivo* evidence for the coordinated activities of Hsp110 and Hsp104 in cytoplasmic and nuclear protein disaggregation. Complexes of Hsp110 and Hsp70 are targeted to protein aggregates and facilitate the recruitment of Hsp104. Hsp104 that has reached the surface of aggregates depends on Hsp110 for productive disaggregation. Thus, Hsp110 play key roles in both the recruitment of Hsp70 and Hsp104 to aggregates as well as the coordinated disaggregation process at the aggregate surface.

## RESULTS

### Sse1 accelerates reactivation of aggregated firefly luciferase in cytosolic lysates.

**FIG 1:**
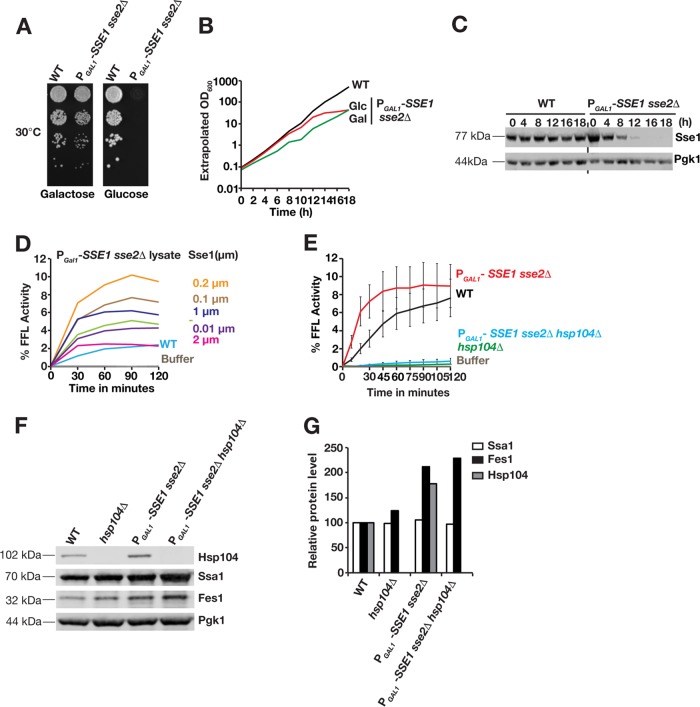
Sse1 accelerates Hsp104-dependent reactivation of chemically aggregated firefly luciferase in cytosolic lysates. (A) Growth of WT and P_*GAL1*_-*SSE1 sse2*Δ strains on media with galactose and glucose as carbon sources. Tenfold serially diluted cell suspensions were plated, and photographs were taken following 3 days of incubation at 30°C. (B) Growth of the strains in panel A was monitored by optical density determinations (extrapolated optical density at 600 nm [OD_600_]) following inoculation of cells in liquid glucose and galactose media. Cells were regularly diluted in prewarmed fresh medium to avoid the effects of nutrient depletion. (C) Western analysis of Sse1 expression levels following the transfer of cells from galactose medium to glucose medium as described above for panel B. Pgk1 functions as a loading control. (D) Reactivation of chemically aggregated firefly luciferase by cytosolic lysates prepared from WT and Sse1-depleted P_*GAL1*_-*SSE1 sse2*Δ cells grown for 18 h in glucose medium. Purified Sse1 was added to the Sse1-depleted lysates at the indicated concentrations. The reactivated fraction of the original firefly luciferase activity (% FFL Activity) was determined by luminescence measurements. (E) Reactivation of chemically aggregated firefly luciferase by cytosolic lysates prepared from WT, *hsp104*Δ, P_GAL1_-*SSE1 sse2*Δ, and P_GAL1_-*SSE1 sse2*Δ *hsp104*Δ cells grown for 18 h in glucose medium. The reactivated fraction of the original firefly luciferase activity (% Activity) was determined by bioluminescence measurements. Error bars indicate standard errors of data from triplicate experiments. (F) Western analysis of the cytosolic lysates used for panel A. (G) Quantification of the relative expression levels in panel B.

We set out to investigate the importance of the yeast Hsp110s Sse1 and Sse2 in the Hsp104-dependent reactivation of aggregated proteins. Previous *in vitro* studies using highly purified setups showed that Sse1 accelerates the reactivation of aggregated firefly luciferase when added to specific mixtures of Hsp40 (21), Hsp70 (Ssa1), and Hsp104 (10, 11). We tested the influence of Sse1 and Sse2 on the reactivation of aggregated firefly luciferase in the context of complete cytosolic lysates, a setup that likely better mirrors the complexity of the cytosolic chaperone system. Depleting the essential Hsp110s from yeast cells by genetically removing *SSE2* and replacing the *SSE1* promoter with a glucose-repressible *GAL1* promoter (P_*GAL1*_-*SSE1 sse2*Δ) resulted in an inability of cells to form colonies on glucose medium (Fig. 1A). We harvested P_*GAL1*_-*SSE1 sse2*Δ cells 18 h after they had been shifted from galactose medium to glucose medium and prepared cytosolic lysates from growth-arrested and Sse1-depleted cells (Fig. 1B and C). Lysate-dependent reactivation was monitored by using aggregated firefly luciferase (see Materials and Methods for details) (20). In this setup, the lysate prepared from control wild-type (WT) cells together with an ATP-regenerating system activated 2.2% of 20 nM aggregated firefly luciferase in 90 min, while the depleted lysate activated 4.7% (Fig. 1D). As expected, reactivation in both types of lysates was strictly dependent on the presence of Hsp104 (Fig. 1E). Western analysis of Sse1-depleted lysates revealed that the proteostasis-perturbing depletion process induced the expression of chaperones, including Hsp104 and the Hsp70 nucleotide exchange factor Fes1 (Fig. 1F and G). Consistent with the notion that the increased chaperone levels induced reactivation activities in the depleted lysates, titration of purified Sse1 into the depleted lysates resulted in an acceleration of reactivation and increased yields beyond what was exhibited by WT reactions (Fig. 1D). While the addition of 0.01 μM Sse1 had little effect, 0.1 μM and 0.2 μM Sse1 accelerated the reactivation reactions and increased the yields from 4.7% to 7.7% and 10.2% after 90 min, respectively. At even higher levels, Sse1 inhibited the reaction, with yields dropping to 6.2% for 1 μM and 2.5% for 2 μM Sse1. Sse1 was previously reported to display similar dose-dependent stimulatory and inhibitory effects on protein folding *in vitro* together with Hsp70 (15). These data suggest that Sse1 is an important cytosolic factor to accelerate Hsp104-dependent disaggregation.

### Sse1 and Sse2 are essential for Hsp104-dependent reactivation of heat-aggregated firefly luciferase in cells.

**FIG 2:**
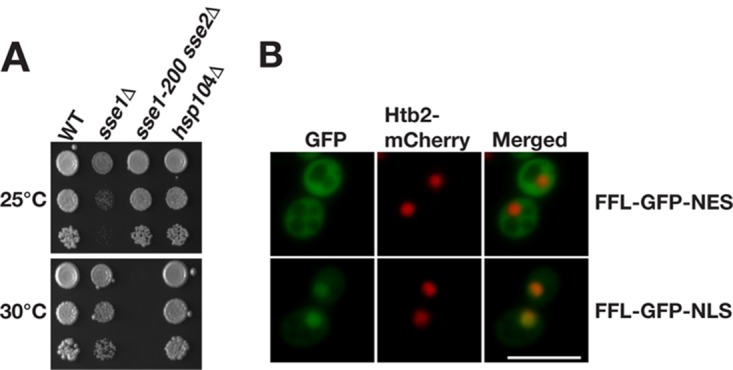
Temperature-sensitive phenotype of *sse1-200 sse2*Δ cells and localization of firefly luciferase reporters. (A) Growth of WT, *sse1*Δ, *sse1-200 sse2*Δ, and *hsp104*Δ strains after 3 days at 25°C and 30°C. (B) Micrographs showing the localization of the firefly luciferase fusion proteins FFL-GFP-NES and FFL-GFP-NLS. Histone 2B fused to mCherry (Htb2-mCherry) functions as a nuclear marker. Bar = 5 μm.

**FIG 3:**
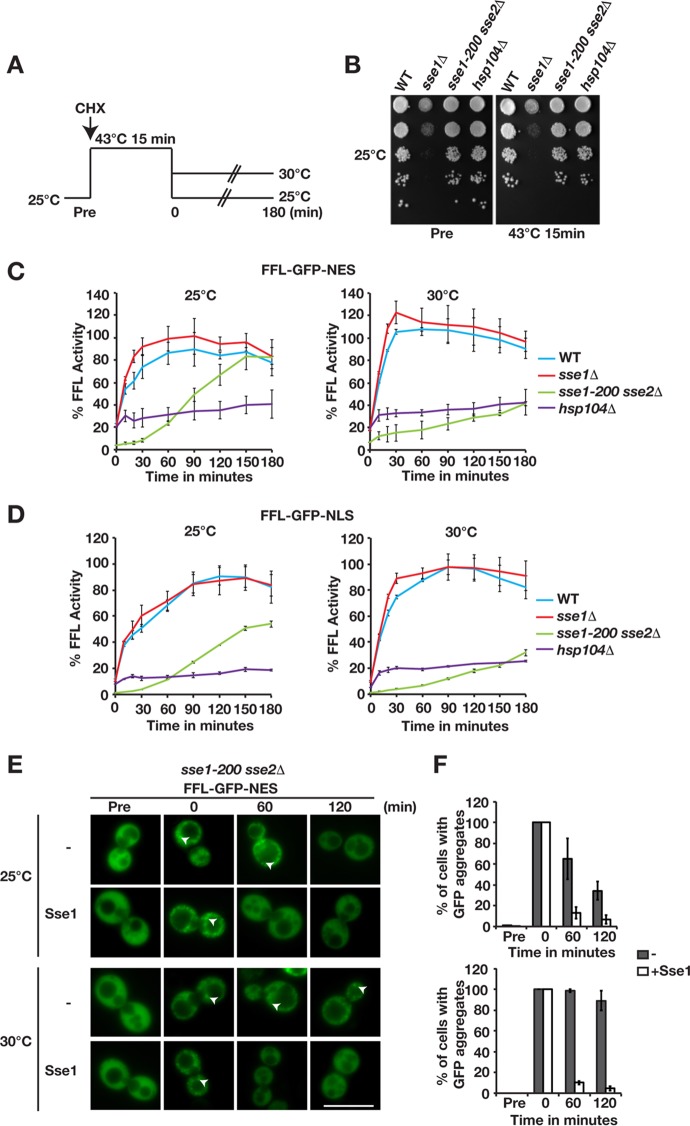
Sse1 and Sse2 are essential for Hsp104-dependent reactivation of heat-aggregated firefly luciferase. (A) Schematic representation of the *in vivo* firefly luciferase reactivation assay. Cells expressing firefly luciferase fused to GFP were pregrown to the logarithmic phase at 25°C. Translation was arrested by the addition of cycloheximide (CHX) followed by 15 min of heat shock at 43°C and recovery at 25°C or 30°C. (B) WT, *sse1*Δ, *sse1-200 sse2*Δ, and *hsp104*Δ cells growing at 25°C (Pre) were subjected to heat shock at 43°C for 15 min, and viability was assessed by the ability of cells to form colonies at 25°C. Photographs were taken 3 days after platting. (C) Reactivation of cytosolic firefly luciferase (FFL-GFP-NES) was monitored by bioluminescence measurements. Error bars represent standard errors of data from triplicate experiments. (D) Reactivation of nuclear firefly luciferase (FFL-GFP-NLS) was monitored as described above for panel C. (E) Micrographs of the disaggregation of FFL-GFP-NES in *sse1-200 sse2*Δ cells transformed with either a centromeric plasmid vector (−) or a derivative that expresses Sse1. Arrowheads show aggregates. Bar = 5 μm. (F) Quantification of the results shown in panel E. Error bars represent standard errors of data from biological triplicates with ≥100 cells for each time point.

We assessed the role of Sse1 and Sse2 in Hsp104-dependent protein disaggregation in cells. The removal of either *SSE1* or *SSE2* was previously shown not to influence the cellular reactivation of heat-aggregated firefly luciferase (19). We reasoned that both members of the essential gene pair have to be simultaneously inactivated to rigorously assess the involvement of Hsp110 (Sse1 and Sse2) in protein disaggregation. Briefly, we isolated a classical temperature-sensitive allele of *SSE1* (*sse1-200*) (see Materials and Methods) that, in the context of *sse2*Δ, allows cells to grow robustly at 25°C and to arrest at 30°C (Fig. 2A). To obtain disaggregation reporters, firefly luciferase was fused to green fluorescent protein (GFP) with either a nuclear export signal (NES) (FFL-GFP-NES) or a nuclear localization signal (NLS) (FFL-GFP-NLS) and was confirmed to be expressed in the cytosol and nucleus, respectively (Fig. 2B). Cells expressing the reporters were grown at 25°C to logarithmic phase, translation was arrested with cycloheximide, and luciferase was inactivated by heat shock at 43°C for 15 min, followed by recovery at 25°C or 30°C (Fig. 3A). Cells subjected to heat shock formed colonies when expanded at 25°C, without a loss of plating efficiency (Fig. 3B). WT and *sse1*Δ cells incubated at either 25°C or 30°C reactivated both cytosolic and nuclear firefly luciferase, with yields close to 100% of the original activity within 90 min (Fig. 3C and D). For *hsp104*Δ cells, no significant gain of luciferase activity was measured over 180 min, demonstrating that disaggregation was Hsp104 dependent. The reactivation of both cytosolic and nuclear firefly luciferase in *sse1-200 sse2*Δ cells that were recovered at the nonpermissive temperature of 30°C was severely defective and did not reach more than 41.1% and 31.9% of the initial activity after 180 min, respectively. Recovery of *sse1-200 sse2*Δ cells at the permissive temperature of 25°C resulted in a delayed reactivation of cytosolic luciferase but with final activity levels comparable to those of WT cells after 180 min (Fig. 3C). Nuclear firefly luciferase in the same strain at 25°C was slowly reactivated over time and reached 53.9% of the initial activity after 180 min (Fig. 3D). Thus, functional Sse1 or Sse2 is required for Hsp104-dependent reactivation of heat-inactivated firefly luciferase in both the cytosol and nucleus.

Next, we microscopically visualized the inactivation and reactivation of FFL-GFP-NES in *sse1-200 sse2*Δ cells that carried a single-copy plasmid vector or a derivative that expressed Sse1 from its endogenous promoter (Fig. 3E). Before heat shock, FFL-GFP-NES was evenly distributed in the cytosol of both cell types. In contrast, heat shock induced massive firefly luciferase aggregation irrespective of the presence or absence of Sse1. Multiple aggregates were present throughout the cytosol following heat shock. Recovery at either 25°C or 30°C showed that the gain of activity was reflected in the reduction of the number of firefly luciferase aggregates. While Sse1-expressing cells efficiently cleared the aggregates at both temperatures within 60 min, 83.2% of the cells lacking Sse1 expression still carried aggregates even after 120 min of incubation at 30°C (Fig. 3F). Incubation of the same cell type at 25°C resulted in the clearance of the aggregates although at reduced rates. Taking the microscopy data together with the activity measurements, we conclude that Sse1 and Sse2 function is essential to enable the Hsp104-dependent reactivation of heat-aggregated firefly luciferase.

### Protein disaggregation in the cytosol and nucleus depends on the Sse1-Hsp70 interaction but not on the intrinsic ATPase activity of Sse1.

**FIG 4:**
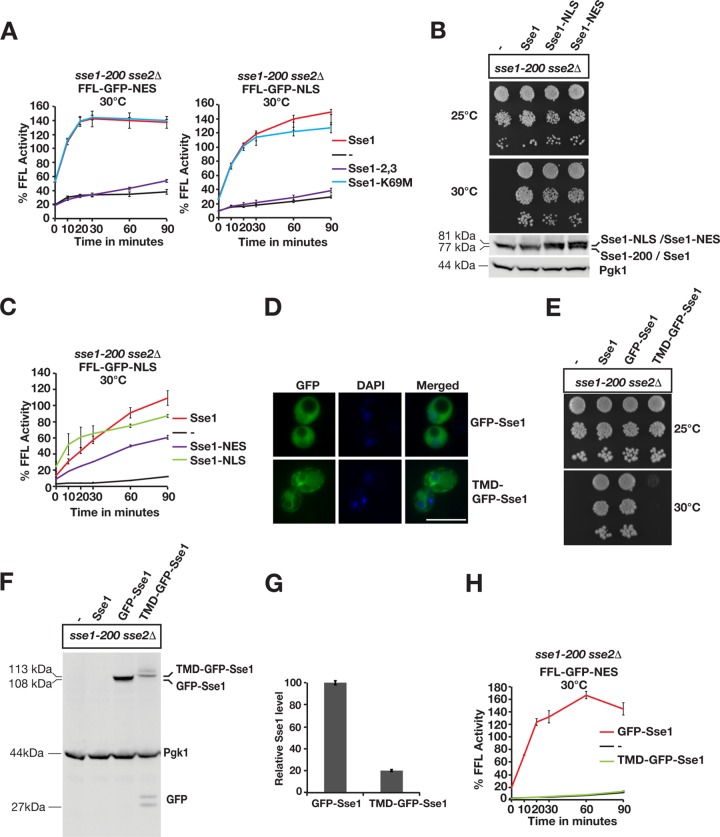
Sse1-dependent protein disaggregation requires interaction with Hsp70 and is compartment specific. (A) Reactivation of cytosolic firefly luciferase (FFL-GFP-NES) and nuclear firefly luciferase (FFL-GFP-NLS) in *sse1-200 sse2*Δ cells was monitored at 30°C after a 15-min heat shock at 43°C by bioluminescence measurements. Cells were transformed with either a centromeric plasmid vector (−) or derivatives that express Sse1, Sse1-2,3, or Sse1-K69M. Error bars represent standard errors of data from triplicate experiments. (B) Growth of *sse1-200 sse2*Δ cells transformed with an empty plasmid vector (−) or derivatives that express Sse1, Sse1-NLS, or Sse1-NES. Photographs of plates were taken 3 days after incubation at 25°C and 30°C. Western blots show the relative expression levels of the Sse1 variants in the strains. (C) Reactivation of nuclear firefly luciferase (FFL-GFP-NLS) was monitored as described above for panel A, but cells were transformed with either a centromeric plasmid vector or derivatives that express Sse1, Sse1-NLS, or Sse1-NES. (D) Fluorescence microscopy image showing the localization of GFP-Sse1 and TMD-GFP-Sse1 in *sse1-200 sse2*Δ cells grown at 25°C. DNA was stained with DAPI (4′,6-diamidino-2-phenylindole) for nuclear localization. (E) Analysis of the growth of *sse1-200 sse2*Δ cells expressing GFP-Sse1 and TMD-GFP-Sse1 as described above for panel B. (F) Western analysis of the strains in panel B. GFP antibodies were used to visualize GFP-Sse1 and TMD-GFP-Sse1. (G) Quantification of the relative expression levels of GFP-Sse1 and TMD-GFP-Sse1 in panel F. (H) Reactivation of cytosolic firefly luciferase (FFL-GFP-NES) was monitored as described above for panel A, but cells were transformed with plasmids that express GFP-Sse1 or TMD-GFP-Sse1. Bar = 5 μm.

We asked if the association of Sse1 with Hsp70 is required for protein disaggregation by employing well-characterized amino acid substitutions (A280T, N281A, N572Y, and E575A) (Sse1-2,3) that specifically impair the interaction with Hsp70 (22). The N572Y and E575A mutations were previously reported to inhibit accelerated disaggregation *in vitro* (11). *In vivo*, the reactivation of aggregated firefly luciferase targeted to either the cytosol or the nucleus was completely abolished in the Sse1-2,3 mutant (Fig. 4A). Firefly luciferase activity exceeded 100% of its initial activity during the recovery period, suggesting that a pool of unfolded luciferase was present in the cells prior to heat treatment. Next, we tested the role of Sse1 ATPase activity in Hsp104-dependent protein disaggregation by analyzing the well-established and hydrolysis-defective Sse1-K69M mutant (23). *In vivo*, we found that the K69M mutation did not impair aggregated firefly luciferase reactivation in the cytosol or nucleus (Fig. 4A). We conclude that intrinsic ATP hydrolysis is not required for Sse1 to support Hsp104-dependent protein disaggregation but that interactions with Hsp70 are.

### Sse1 activity is required in the same compartment for efficient disaggregation.

We asked if Sse1 has to localize to the same compartment as the aggregated proteins to support their reactivation. Sse1 is predominantly localized to the cytosol, with lower expression levels in the nucleus (Fig. 4D). We fused NESs and NLSs to the C terminus of Sse1 to decrease and increase the levels of Sse1 in the nucleus and monitored the reactivation of nuclear firefly luciferase. Both Sse1-NES and Sse1-NLS supported the essential Hsp110 functions required for the growth of yeast cells (Fig. 4B). The exclusion of Sse1 from the nucleus (Sse1-NES) resulted in decreased rates of reactivation of nuclear firefly luciferase aggregates (Fig. 4D). In contrast, targeting of Sse1 to the nucleus (Sse1-NLS) accelerated reactivation to rates comparable to, or higher than, those of WT Sse1. The residual disaggregation activity in cells that expressed Sse1-NES could be the result of either an incomplete nuclear export of Sse1-NES or nucleotide exchange of Hsp70 in the cytosol. Our data suggest that Sse1 has to reside in the nuclear compartment to efficiently support nuclear disaggregation.

We limited the movement of Sse1 by anchoring it to the cytosolic leaflet of the endoplasmic reticulum by fusing a single-pass transmembrane domain (TMD) carrying GFP to its N terminus (Fig. 4D). Fusing GFP alone to the N terminus of Sse1 (GFP-Sse1) did not impair the functions required for growth, but immobilizing the protein to the membrane did (TMD-GFP-Sse1) (Fig. 4E to G). Similarly, anchored Sse1 did not support any reactivation of cytosolic firefly luciferase aggregates (Fig. 4H). The data from these experiments suggest that freely diffusible Sse1 is required in the same compartment as the Hsp104-dependent disaggregation reaction.

### Recruitment of Hsp70 to aggregates depends on interaction with Sse1.

**FIG 5:**
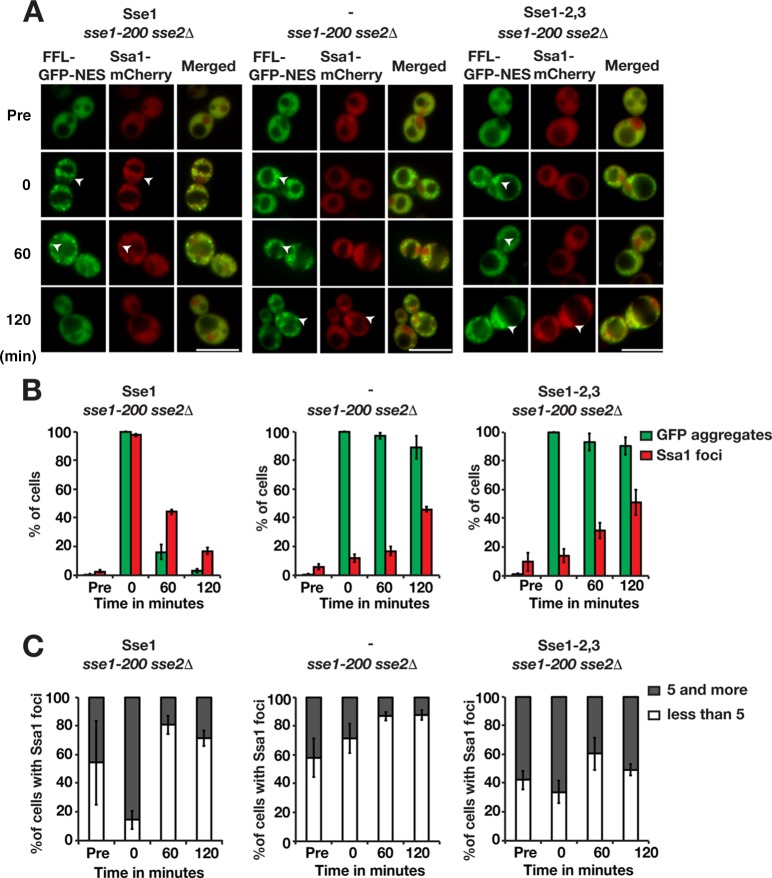
Sse1 is required for the efficient recruitment of Hsp70 to protein aggregates. (A) Fluorescence microscopy images of FFL-GFP-NES and Ssa1-mCherry in *sse1-200 sse2*Δ cells transformed with an empty plasmid vector control (−) or derivatives that express Sse1 or Sse1-2,3. Cells were pregrown at 25°C (Pre), heat shocked at 43°C for 15 min, and maintained at 30°C as outlined in the legend to Fig. 3A. Arrowheads show aggregates. Bars = 5 μm. (B) Quantification of the fraction of cells in panel A with FFL-GFP-NES aggregates and Ssa1-mCherry foci. Error bars represent standard errors from biological triplicates with ≥100 cells for each time point. (C) Percentage of Ssa1 aggregate-harboring cells with 1 to 4 and ≥5 aggregates. Shown are quantifications of the results in panel A.

Hsp70 is targeted to aggregates by Hsp40 (24, 25). We investigated the importance of Sse1 for the recruitment of Hsp70 to aggregates by monitoring Ssa1-mCherry localization after heat shock. In cells expressing functional Sse1, Ssa1 aggregates were detectable in 2.4% of the cells before heat shock, and 54.1% of these cells contained <5 aggregates (Fig. 5). Immediately after heat shock, 97.6% of the cells contained Ssa1 aggregates that colocalized with FFL-GFP-NES aggregates, and of those cells, 85.4% harbored ≥5 Ssa1 aggregates. The fraction of cells with Ssa1 aggregates and the number of aggregates in the cells decreased during the recovery phase, with 16.8% of the cells containing aggregates after 120 min. In contrast, Ssa1 recruitment to the aggregates was severely impaired in cells lacking Sse1 and in Sse1-2,3-expressing cells. Specifically, only 11.7% and 14.1% of the cells, respectively, scored positive for Ssa1 aggregates directly following heat shock. Following recovery for 120 min, the fractions of cells containing Ssa1 aggregates had increased to 45.9% for cells lacking functional Sse1 and 50.9% for Sse1-2,3-expressing cells. Quantification showed that the number of Ssa1 aggregates in Sse1 mutant cells changed little during the recovery phase (Fig. 5B and C). This finding is consistent with the finding that *sse1*Δ cells exhibit decreased Q-body (peripheral aggregate) dynamics (26). Our data show that Sse1 is important for the recruitment of Ssa1 to aggregates, which depends on the ability of Sse1 to associate with Hsp70.

### Sse1 facilitates Hsp104 recruitment to protein aggregates.

**FIG 6:**
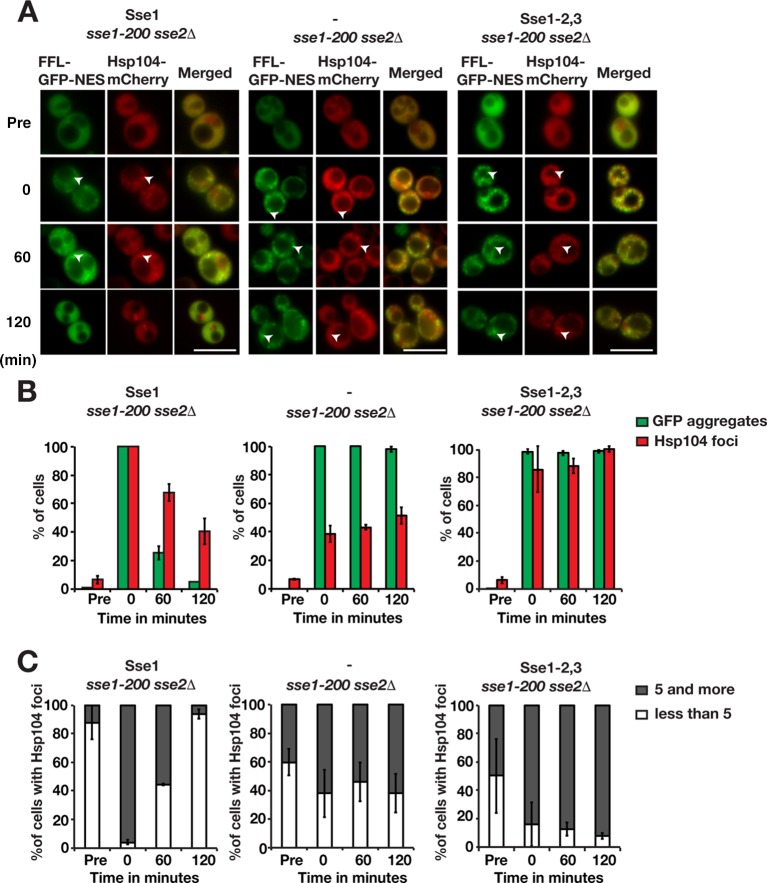
Sse1 is required for the efficient recruitment of Hsp104 to protein aggregates. (A) Fluorescence microscopy images of FFL-GFP-NES and Hsp104-mCherry in *sse1-200 sse2*Δ cells transformed with an empty plasmid vector control (−) or derivatives that express Sse1 or Sse1-2,3. Cells were pregrown at 25°C (Pre), heat shocked at 43°C for 15 min, and maintained at 30°C as outlined in the legend to Fig. 3A. Arrowheads show aggregates. Bars = 5 μm. (B) Quantification of the fraction of cells in panel A with FFL-GFP-NES aggregates and Hsp104-mCherry foci. Error bars represent standard errors from biological triplicates with ≥100 cells for each time point. (C) Percentage of Hsp104 aggregate-harboring cells with 1 to 4 and ≥5 aggregates. Shown are quantifications of the results shown in panel A.

We investigated if Sse1 is important for the recruitment of Hsp104 to protein aggregates by monitoring Hsp104-mCherry microscopically in heat-shocked *sse1-200 sse2*Δ cells. In cells complemented with Sse1 expression, the behavior of Hsp104 mirrored what was observed for Ssa1 (Fig. 6). Following heat shock, 100% of the cells contained Hsp104 aggregates, with 95.9% having ≥5 aggregates and many colocalizing with FFL-GFP-NES aggregates. During the recovery phase, FFL-GFP-NES and Hsp104 aggregates were cleared from the cells, and after 120 min, 40.5% of the cells contained Hsp104 aggregates, 93.8% of which had <5 foci. Hsp104 was previously shown to be associated with both IPODs (insoluble protein deposits) and the JUNQ/INQ (juxtanuclear quality control compartment/intranuclear quality control compartment) (27–29). Consistent with the formation of a JUNQ/INQ during recovery after heat shock, we observed Hsp104 foci associated with the nucleus after 120 min. In contrast, in cells with no functional Sse1, the recruitment of Hsp104 to the aggregates immediately after heat shock was impaired, with only 38.5% of cells containing Hsp104 peripheral aggregates. During the recovery phase, the number of *sse1-200 sse2*Δ cells containing Hsp104 aggregates increased somewhat to 51.4% after 120 min. Notably, the number of peripheral aggregates did not decrease significantly during the recovery phase, indicating a general problem in Hsp104-dependent disaggregation (Fig. 6B and C). Thus, Sse1 activity is important for both the recruitment of Hsp104 to aggregates and disaggregation activity once Hsp104 has been recruited to the aggregate.

Surprisingly, the recruitment of Hsp104 to aggregates was not strictly dependent on the ability of Sse1 to interact with Hsp70, since 85.8% of cells expressing Sse1-2,3 contained Hsp104 aggregates directly after heat shock (Fig. 6). Nevertheless, the recruited Hsp104 was inefficient in solubilizing the aggregates: 100% of the cells retained Hsp104 aggregates after 120 min, and most of these cells retained ≥5 aggregates. It is unclear at present if this finding simply reflects a residual activity of the Sse1-2,3 mutant or that Hsp104 recruitment does not require a complex between Hsp70 and Hsp110.

### Hsp70 nucleotide exchange activity is not sufficient for efficient protein disaggregation.

**FIG 7:**
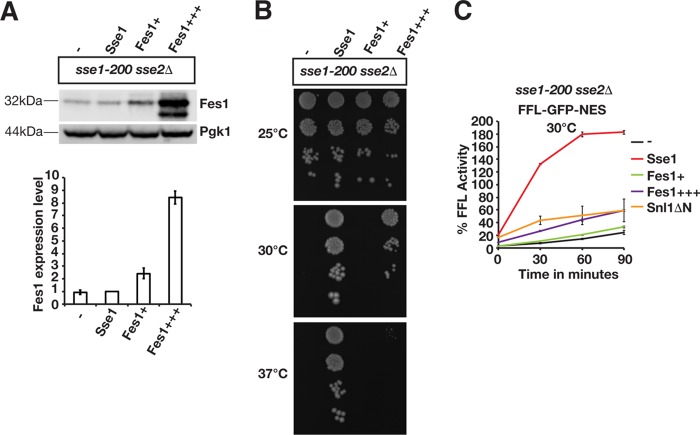
Overexpressed Fes1 and Snl1ΔN do not replace the function of Sse1 and Sse2 in the reactivation of heat-aggregated firefly luciferase. (A) Western analysis of *sse1-200 sse2*Δ cells transformed with an empty vector control (−) or plasmids that express Sse1 or Fes1 at lower (Fes1^+^) or higher (Fes1^+++^) levels. Fes1 expression levels were determined relative to those of the strain transformed with the Sse1-expressing plasmid (bottom). B) Growth of *sse1-200 sse2*Δ cells transformed with an empty plasmid vector (−) or derivatives that express Sse1, Fes1^+^, or Fes1^+++^. (C) Reactivation of heat-aggregated cytosolic firefly luciferase (FFL-GFP-NES) in Fes1- and Snl1ΔN-overexpressing strains was monitored by bioluminescence measurements. Error bars represent standard errors of data from triplicate experiments.

Overexpression of Hsp70 nucleotide exchange factors that are structurally unrelated to Sse1 and Sse2 was previously reported to suppress certain Hsp110 phenotypes (15, 30–33). We tested if increased nucleotide exchange activity suppressed the *sse1-200 sse2*Δ protein disaggregation phenotype by overexpressing the nucleotide exchange factors Fes1 and Snl1ΔN (34–36). Fes1 was overexpressed from two different plasmids, resulting in levels that were increased 2.4-fold (this lower level of overexpression is indicated as Fes1^+^) and 8.4-fold (Fes1^+++^), respectively (Fig. 7A). Consistent with data from a previous study (15), Fes1 overexpression (this higher level overexpression is indicated as Fes1^+++^) supported the growth of *sse1-200 sse2*Δ cells at 30°C but was unable to complement the phenotype at 37°C (Fig. 7B). Fes1 overexpression at the lower level (Fes1^+^) resulted in 33.2% firefly luciferase activity after 90 min, which was not significantly different from that of control cells lacking functional Sse1 (Fig. 7C). Modest reactivation was observed with the higher level of Fes1 overexpression, yielding 59% of the original activity after 90 min. A similar inefficient reactivation was obtained when Snl1ΔN was overexpressed. In contrast, the Sse1-complemented strain reactivated 182.5% of the initial firefly luciferase activity after 90 min. Thus, higher levels of other Hsp70 nucleotide exchange factors only partially improve Hsp104-dependent protein reactivation in the absence of Sse1 and Sse2. These data suggest that Sse1 and Sse2 function differently from other Hsp70 nucleotide exchange factors in protein disaggregation.

### Sse1 is recruited to protein aggregates depending on its interaction with Hsp70.

**FIG 8:**
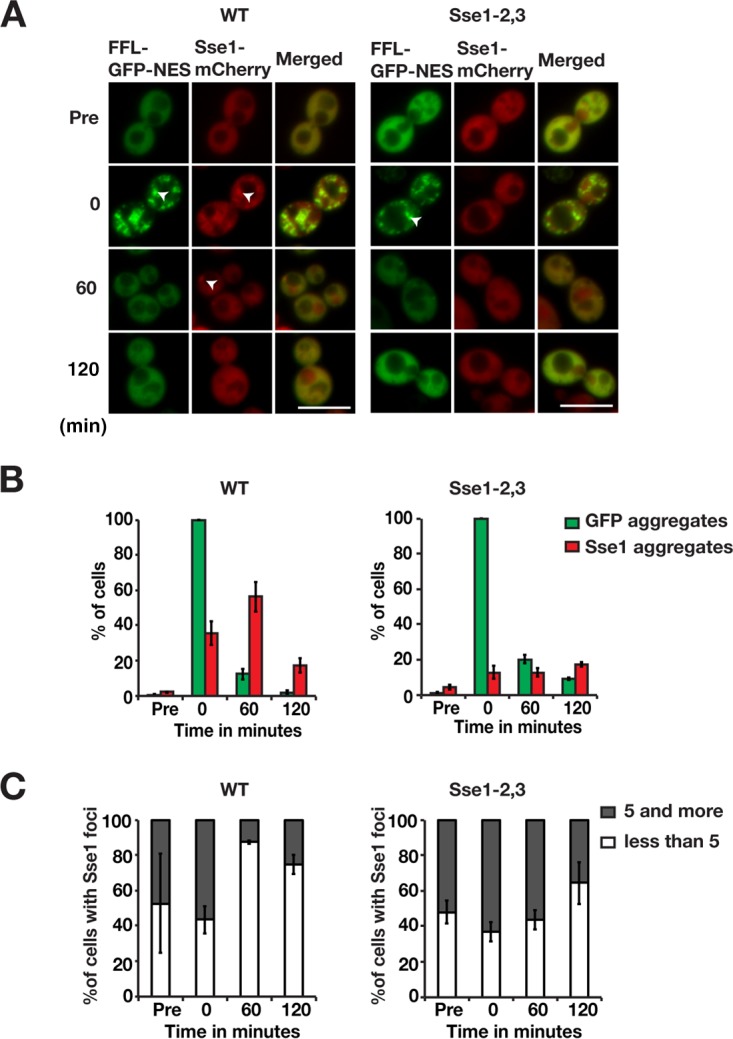
Sse1 is targeted to protein aggregates depending on its association with Hsp70. (A) Fluorescence microscopy images of cells expressing FFL-GFP-NES and Sse1-mCherry (left) or Sse1-2,3–mCherry (right) following heat shock. Cells were pregrown to logarithmic phase at 25°C (Pre), heat shocked for 15 min at 43°C, and allowed to recover at 30°C (0, 60, and 120 min). Arrowheads show aggregates. Bars = 5 μm. (B) Quantification of the fraction of cells in panel A with FFL-GFP-NES aggregates and Sse1-mCherry foci. Error bars represent standard errors from biological triplicates with ≥100 cells for each time point. (C) Percentage of Sse1 aggregate-harboring cells with 1 to 4 and ≥5 aggregates. Shown are quantifications of the results shown in panel A.

Taking all the above-described results into account, we considered the possibility that Sse1 directly engages protein aggregates. It was recently shown that Sse1 is associated with amyloid fibrils *in vitro* (37). We chromosomally fused mCherry to the C terminus of Sse1 (Sse1-mCherry) and monitored the protein microscopically together with cytosolic firefly luciferase (FFL-GFP-NES) in translating cells subjected to heat shock for 15 min at 43°C. Sse1, together with FFL-GFP-NES, was evenly localized throughout the cytosol before heat shock (Fig. 8A). Directly following heat shock, 35.6% of the cells contained Sse1 aggregates that colocalized with aggregated FFL-GFP-NES (Fig. 8A and B). During recovery at 30°C, the fraction of cells with Sse1 aggregates increased to 56.2% after 60 min, eventually decreasing to 17.4% after 120 min. Of the cells with detectable Sse1 aggregates, 56.5% carried ≥5 Sse1 aggregates immediately after heat shock, and the number of aggregates was reduced over time so that 12.5% contained ≥5 Sse1 aggregates after 60 min (Fig. 8C). We observed that after 60 min of recovery, Sse1 in most cells formed a single aggregate adjacent to the vacuole, and this type of aggregate was cleared after 120 min. Thus, following heat shock, Sse1 is targeted to protein aggregates in a time frame relevant for protein disaggregation.

We next asked if the association of Sse1 with protein aggregates was dependent on an interaction with Hsp70. The Hsp70-binding mutant Sse1-2,3 was fused to mCherry (Sse1-2,3–mCherry) and expressed from the chromosome. Despite the fact that Sse1-2,3 does not support firefly luciferase disaggregation, the presence of functional Sse2 in these cells enabled unperturbed disaggregation of cytosolic firefly luciferase following heat shock (Fig. 8). In contrast to wild-type Sse1, the Hsp70-binding mutant was not recruited to aggregates following heat shock. Only 12.8% of the cells displayed Sse1-2,3 aggregates after heat shock, and this value stayed almost constant over 120 min of recovery at 30°C (Fig. 8A and B). The number of Sse1-2,3 aggregates in the few cells that scored positive did not change much during the recovery phase (Fig. 8C). Thus, Sse1 requires an interaction with Hsp70 to associate with protein aggregates. Accordingly, these data suggest that Sse1 and Sse2 function in complexes with Hsp70 at the aggregate surface to support Hsp104-dependent protein disaggregation.

## DISCUSSION

**FIG 9:**
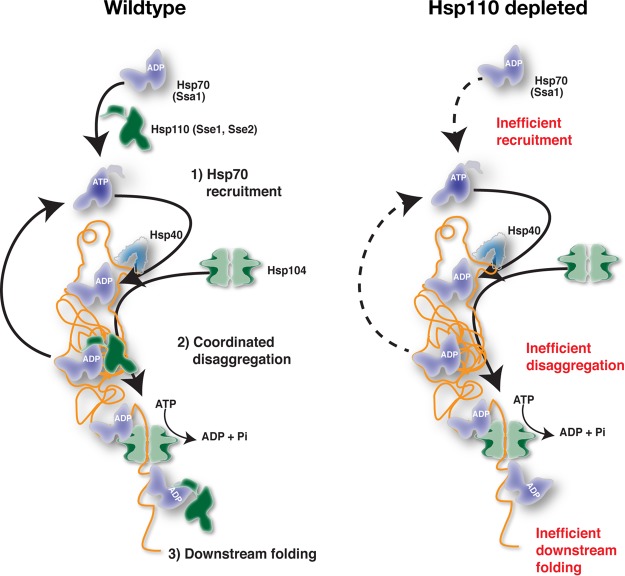
Model for functions of Hsp110 in protein disaggregation. See Discussion for details.

We present evidence that Hsp110 is essential for the disaggregation of proteins in the cytosol and nucleus of yeast cells. To summarize how Hsp110 facilitates Hsp104-dependent disaggregation, we present a model in Fig. 9. In WT cells, Hsp110 impacts Hsp104-dependent disaggregation at the levels of (i) Hsp70 recruitment to aggregates, (ii) Hsp110-Hsp104 coordinated disaggregation, and (iii) protein folding downstream of Hsp104. Each of these steps is discussed separately below in the context of the current understanding of Hsp104 function (7, 38, 39).

For recruitment to aggregates, Hsp70 associates with exposed hydrophobic peptide segments and Hsp40 in its ATP-bound form. The association of Hsp40 and the substrate with Hsp70 accelerates ATP hydrolysis, resulting in the ADP-bound closed conformation of Hsp70. Hsp110, the most abundant Hsp70 nucleotide exchange factor in the cell, recycles Hsp70 back to the ATP-bound form and thereby increases the available pool of free Hsp70. Hence, Hsp70-ATP is predicted to rapidly become limiting upon Hsp110 inactivation and thus to impair Hsp70 recruitment to aggregates. Indeed, we observed that in the absence of Hsp110 activity, Ssa1 was not efficiently recruited to the aggregates directly following heat shock. Consistent with the notion that Ssa1-ATP was limiting under these conditions, prolonged incubation over 120 min resulted in slow but detectable Ssa1 recruitment. Further support came from the defective recruitment of Ssa1 in cells expressing Sse1-2,3, a mutant that cannot interact with Hsp70 and accelerate nucleotide exchange. Nevertheless, the role of Hsp110 in Hsp104-dependent disaggregation may not simply be to maintain sufficient cytosolic or nuclear levels of Hsp70-ATP since the overexpression of other Hsp70 nucleotide exchange factors (Fes1 and Snl1ΔN) in the absence of Hsp110 only modestly improved the reactivation of aggregated firefly luciferase. Independent of any potential additional activities, a key function of Hsp110 is to function as a nucleotide exchange factor for Hsp70 resulting in the efficient recruitment of Hsp70-ATP to aggregates.

Our data suggest that Hsp110 is involved in the disaggregation process at the aggregate surface. Sse1 itself is recruited to aggregates depending on its interaction with Hsp70. Tethering of Sse1 to the endoplasmic reticulum membrane abolished Hsp104-dependent disaggregation in the cytosol, and similarly, export of Sse1 from the nucleus impaired reactivation in the nucleoplasm. The function of Sse1 at aggregates likely involves nucleotide exchange interactions with Hsp70 that enable the remodeling of the aggregate surface. According to this scenario, the dynamic Hsp70-Hsp110 complexes contribute to the overall disaggregation output by working on the aggregate surface side by side with Hsp70-Hsp104. Hsp70-Hsp110 would facilitate the conformational remodeling of aggregated proteins to enable their rapid translocation through the central channel of Hsp104. For example, Hsp70-Hsp110 may destabilize protein structures resistant to the pulling force of Hsp104. Hsp110 could also contribute to disaggregation by releasing Hsp70 that sterically hinders the translocation of the polypeptide through Hsp104. Consistent with the notion of a role of Hsp110 in disaggregation at the aggregate surface, we observed that Hsp104 was recruited to aggregates in 40% of cells that lacked functional Hsp110 immediately after heat shock but still failed to disaggregate. Thus, Hsp104 is stalled in futile translocation attempts awaiting Hsp70-Hsp110. This reasoning leads to the intriguing conclusion that Hsp70-Hsp110 disaggregation activity is coordinated with Hsp104 activity.

Hsp110 may also contribute to protein folding downstream of Hsp104. Translocation via Hsp104 results in the complete unfolding of the protein and necessitates *ab initio* folding of the entire polypeptide, similarly to what happens following translation. Since Hsp70 and presumably also Hsp110 play key roles in folding linked to translation, Hsp104-translocated proteins may depend on Hsp70-Hsp110 to attain their native conformations and, if folding fails, perhaps aggregate again. Still, the involvement of a chaperone downstream of Hsp104 translocation is a poorly explored subject (7, 40, 41).

The strict requirement for Hsp110 in Hsp104-dependent disaggregation in cells is not fully reflected in reconstituted disaggregation *in vitro*. Minimally, Hsp104 titrated with representatives of each of the Hsp70 and Hsp40 classes is sufficient for *in vitro* disaggregation activity (11, 20). Similarly, we found that complete cytosolic lysates highly depleted of Sse1 and Sse2 supported the *in vitro* disaggregation of aggregated firefly luciferase. Still, titration of recombinant Sse1 to the depleted lysates greatly accelerated reactivation and final yields of firefly luciferase activity. The accelerated Hsp104-dependent disaggregation that we observe is consistent with observations from previous reports employing minimally reconstituted systems of Hsp104-independent disaggregation (10, 11). While *in vitro* disaggregation experiments are powerful tools for characterizing the basic activities of Hsp104, they are based on specific conditions that enable the rapid reactivation of model substrates *in vitro*. For example, cellular Hsp70 substrates are usually not present at high levels during *in vitro* disaggregation, potentially making the system less dependent on Hsp110 nucleotide exchange activity. However, by using complex cytosolic lysates, which presumably contain many of the cellular Hsp70 substrates, we still observe Hsp110-independent disaggregation of exogenously added model substrates. Alternatively, the *in vitro* model substrates may have different characteristics than those *in vivo*. Specifically, heat-aggregated firefly luciferase displays experiment-to-experiment variability when used for *in vitro* disaggregation, while instead, urea denaturation gives highly reproducible results (20). It is therefore likely that firefly luciferase that has aggregated in the cellular milieu may display unique characteristics in disaggregation. It remains to be elucidated if such substrate-specific properties may underlie the difference in the dependencies on Hsp110 *in vitro* and *in vivo*.

Our finding that Hsp110 is essential for disaggregation in yeast cells raises the question of whether Sse1 and Sse2 function as ATP-consuming disaggregases that, analogously to Hsp70, rely on nucleotide-regulated allosteric conformational changes. Previous amide hydrogen exchange experiments demonstrated that Sse1 does not undergo allosteric conformational changes in response to ATP hydrolysis (42). However, mammalian Hsp110 appears to consume ATP when it is disaggregating proteins together with Hsp40 (8), and the ATP hydrolysis-defective Sse1-K69M mutant has been reported to be inactive in accelerating Hsp104-independent protein disaggregation (11). In contrast, a later study found that Sse1-K69M was functional in accelerating *in vitro* disaggregation (10). In our *in vivo* assays, Sse1-K69M supported the Hsp104-dependent reactivation of aggregated firefly luciferase as well as the wild-type protein. Thus, our results lend support to the notion that Sse1 does not rely on nucleotide-regulated allosteric conformational changes to facilitate protein disaggregation in cells. Instead, Sse1-ATP contributes to protein disaggregation by forming complexes with Hsp70. This does not rule out that Hsp110 interacts with proteins as a chaperone.

We have not directly addressed the role of Hsp110 in amyloid aggregate remodeling, but since Hsp104 also engages this type of aggregate, Hsp110 is likely involved. Hsp110 has been implicated in the formation of [*PSI*^+^] prions (43, 44) and influences their propagation by enhancing the nucleation step (30). A recent study suggests that Hsp104 and Hsp110 regulate prion fibril length by directly engaging [*PSI*^+^] fibrils (37). *In vitro*, human Hsp70, along with Hsp110 and Hsp40 (DNAJB1) (a class B J-protein), disassembles amyloid aggregates by fragmentation and depolymerization (45). Thus, the coordinated activities of Hsp110 and Hsp104 in disaggregation may generally apply to both amorphous and amyloid aggregates.

The importance of Hsp110 for Hsp104-dependent disaggregation in yeast also has potential therapeutic relevance. Hsp104 has been proposed to be useful as a therapeutic chaperone for the treatment of protein misfolding diseases and neurodegenerative disorders (46–50). Coordinated disaggregation by Hsp110 and Hsp104 in yeast suggests that the interplay between these chaperones should be considered before Hsp104 is introduced into human cells for therapeutic purposes.

## MATERIALS AND METHODS

### Strains and plasmids.

The yeast strains and plasmids used in this study are listed in Tables 1 and 2, respectively. To obtain *sse1-200*, *SSE1* was amplified by error-prone PCR using *Taq* DNA polymerase in the presence of Mn^2+^ and tilted (1 mM dCTP-dTTP and 0.2 mM dATP-dGTP) deoxynucleoside triphosphate (dNTP) concentrations (51). Following cloning of the library by the cotransformation of strain CAY1031 with EcoRI-restricted pCA502 and the PCR product, the *sse1-200* allele (mutations S405P, P453L, E463G, Y496C, E511G, K571R, and M604T) was selected based on its ability to confer robust growth at 25°C and growth arrest at 30°C. The *sse1-200* allele was chromosomally integrated into the *his3*Δ*1* locus under the control of its endogenous promoter for further characterization. Yeast cells were grown in standard yeast-peptone-dextrose (YPD) medium or in synthetic complete (SC) medium to select for strains transformed with plasmids.

**TABLE 1:** Yeast strains

Strain	Genotype	Figure(s)	Reference or source
CAY1015	*MAT***a** *his3*Δ*1 leu2*Δ*0 ura3*Δ*0*	1, 2A, 3B–D	34
CAY1039	*MAT***a** *his3*Δ*1 leu2*Δ*0 met15*Δ*0 ura3*Δ*0 sse2*Δ::*hphMX4 SSE1*Δ*P*::*kanMX*-*PGAL*	1	This work
CAY1051	*MAT***a** *his3*Δ*1 leu2*Δ*0 met15*Δ*0 ura3*Δ*0 sse2*Δ::*hphMX4 SSE1*Δ*P*::*kanMX*-*PGAL hsp104*::*natMX4*	1E–G	This work
CAY1337	*MAT***a** *leu2*Δ*0 met15*Δ*0 ura3*Δ*0 sse1*Δ::*hphMX4 sse2*Δ::*kanMX4 his3*Δ*1*::[*his3*Δ::his6]-*sse1*-*CT3*	2A, 3B–E, 4, 7	This work
CAY1031	*MAT***a** *his3*Δ*1 leu2*Δ*0 ura3*Δ*0 sse1*Δ::*kanMX*	2A, 3B–D	34
CAY1054	*MAT***a** *his3*Δ*1 leu2*Δ*0 ura3*Δ*0 hsp104*Δ::*natMX*	2A, 3B–D	This work
CAY1343	*MAT***a** *leu2*Δ*0 met15*Δ*0 ura3*Δ*0 sse1*Δ::*hphMX4* *sse2*Δ::*kanMX4 his3*Δ*1*::[*his3*Δ::his6]-*sse1-CT3 HSP104-ymCherry-nat*^R^	6	This work
CAY1361	*MAT***a** *leu2*Δ*0 met15*Δ*0 ura3*Δ*0 sse1*Δ::*hphMX4 sse2*Δ::*kanMX4 his3*Δ*1*::[*his3*Δ::his6]-*sse1-CT3 SSA1::ymCherry-PTEF-nat*^R^	5	This work
CAY1357	*MAT***a** *his3*Δ*1 leu2*Δ*0 ura3*Δ*0 SSE1-ymCherry-PTEF-kan*^R^	8	This work
CAY1362	*MAT***a** *his3*Δ*1 leu2*Δ*0 ura3*Δ*0 SSE1-2,3 ymCherry-PTEF-kan*^R^	8	This work
CAY1259	*MAT***a** *his3*Δ*1 leu2*Δ*0 ura3*Δ*0 HTB2::ymCherry-kanMX*	2B	This work

**TABLE 2:** Plasmids

Plasmid	Description	Figure(s)	Reference or source
pCA502	*CEN/ARS HIS3*; Ap^r^	3E and F; 4A–C, E, F, and H; 5–7	42
pCA503	*SSE1 CEN/ARS HIS3*; Ap^r^	3E and F; 4A–C, E, and F; 5–7	42
pCA923	P_TDH3_-Luciferase-GFP-NES (PKI) CEN/ARS URA3; Ap^r^	2B; 3C, E, and F; 4A and H; 5; 6; 7C; 8	This work
pCA924	P_TDH3_-Luciferase-GFP-NLS (PKI) CEN/ARS URA3; Ap^r^	2B, 3D, 4A and C	This work
pCA630	pCA503 Sse1-K69M *CEN/ARS HIS3*; Ap^r^	4A	This work
pCA899	Sse1_A280T,N281T,E572Y,E575A_ *CEN/ARS HIS3*; Ap^r^	4A, 5, 6	This work
PCA870	pCA502 *FES1* (entire locus)	7	43
PCA970	Sse1-NES *CEN/ARS HIS3*; Ap^r^	4B and C	This work
pJK010	Sse1-NLS *CEN/ARS HIS3*; Ap^r^	4B and C	This work
pJK049	TMD-ysfGFP-Sse1 2μ *His3*; Ap^r^	4D–H	This work
pJK001	ysfGFP-Sse1 *CEN/ARS HIS3*; Ap^r^	4D–H	This work
P2H-GPD-FES1	P_GPD_-*FES1* 2μ *HIS3*; Ap^r^	7	This work
P2H-GPD-Snl1ΔN	P_GPD_-*SNL1*ΔN 2μ *LEU2*; Ap^r^	7C	This work

### Firefly luciferase reactivation in cell lysates.

Reactivation of firefly luciferase from aggregates was performed as outlined previously (20). Firefly luciferase was chemically denatured in 8 M urea–10 mM dithiothreitol (DTT) at 10 μM and diluted in ice-cold refolding buffer (RFB) (40 mM HEPES-KOH [pH 7.5], 150 mM KCl, 10 mM MgCl_2_, 10 mM DTT, and 1 mM phenylmethylsulfonyl fluoride [PMSF]) to a final concentration of 200 nM, and aliquots of denatured firefly luciferase (dFFL) were snap-frozen in liquid nitrogen and stored at −80°C. For lysate preparation, cells were grown in YPD for 18 h at 30°C and harvested by centrifugation. Frozen cells were ground to a powder in 2-ml screw-cap Eppendorf tubes fitted with 7.1-mm steel balls by using a Mini-Beadbeater (Biospec Products, Inc., Bartlesville, OK) at 2,500 rpm for 30 s and thawed in ice-cold RFB. Following ultracentrifugation at 100,000 × *g* for 30 min, protein concentrations were determined by using the Bradford assay (52). Reactivation of 20 nM dFFL in RFB supplemented with 2 mg/ml yeast lysate, 50 mM phosphate, 5 mM ATP, 20 mM creatine phosphate, and 20 μg/ml creatine phosphokinase was monitored at 25°C by mixing aliquots of the reactivation mixture with d-luciferin and ATP in an Orion II microplate luminometer (Berthold, Pforzheim, Germany). Reactivation mixtures were supplemented with purified Sse1 (53).

### Western analysis.

Total protein samples were prepared from cells grown to logarithmic phase by NaOH treatment followed by trichloroacetic acid (TCA) precipitation (54). Western analysis was performed as described previously (55). Briefly, equal amounts of SDS-solubilized protein were subjected to SDS-PAGE and Western blotting. Nitrocellulose membranes were incubated with the following primary antibodies for 1 h at room temperature: anti-Fes1 (rabbit serum; 1:5,000), anti-Ssa1 (rabbit serum; 1:50,000), anti-Hsp104 (yeast polyclonal antibody [Enzo Lifescience, Farmingdale, NY]; 1:1,000), and anti-Sse1 (rabbit serum; 1:50,000). Following incubation of secondary antibody for 1 h, membranes were developed by using the Odyssey Fc near-infrared fluorescence detection instrument (Li-Cor Biosciences, Lincoln, NE) and analyzed by using Image Studio Lite software.

### Fluorescence microscopy.

Live images were taken by using a Zeiss Axiovert 200 M inverted fluorescence microscope (Carl Zeiss, Jena, Germany) with a Plan-apochromatic 63×/1.4-numerical-aperture oil immersion lens, a DG4 light source (Sutter Instruments, Novato, CA) equipped with an AxioCam MRm camera (Carl Zeiss), and SlideBook 5.0 software (Intelligent Imaging Innovations, GmbH, Göttingen, Germany). Images were acquired and processed by using SlideBook Reader software. Image quantification was done by using ImageJ software (National Institutes of Health, Bethesda, MD).

### *In vivo* firefly luciferase reactivation assay.

Cells expressing firefly luciferase (FFL-GFP-NES and FFL-GFP-NLS) were grown to logarithmic phase at 25°C. Cultures were treated with cycloheximide at 100 mg/liter and heat shocked at 43°C for 15 min in a water bath, and reactivation at 25°C or 30°C was monitored by using an Orion II microplate luminometer (Berthold, Germany) with 100 μl of logarithmically growing cells and 50 μl d-luciferin at 455 μg/ml as described previously (56).
